# Tissue pO_2_ distributions in xenograft tumors dynamically imaged by Cherenkov-excited phosphorescence during fractionated radiation therapy

**DOI:** 10.1038/s41467-020-14415-9

**Published:** 2020-01-29

**Authors:** Xu Cao, Srinivasa Rao Allu, Shudong Jiang, Mengyu Jia, Jason R. Gunn, Cuiping Yao, Ethan P. LaRochelle, Jennifer R. Shell, Petr Bruza, David J. Gladstone, Lesley A. Jarvis, Jie Tian, Sergei A. Vinogradov, Brian W. Pogue

**Affiliations:** 1 Thayer School of Engineering, Dartmouth College, Hanover, NH USA; 2 Engineering Research Center of Molecular and Neuro Imaging of Ministry of Education, School of Life Science and Technology, Xidian University, Xi’an, Shaanxi China; 3 Department of Biochemistry and Biophysics, Perelman School of Medicine, University of Pennsylvania, Philadelphia, PA USA; 4 Department of Chemistry, School of Arts and Sciences, University of Pennsylvania, Philadelphia, PA USA; 5 Norris Cotton Cancer Center, Dartmouth-Hitchcock Medical Center, Lebanon, NH USA; 6 Key Laboratory of Biomedical Information Engineering of Ministry of Education, Institute of Biomedical Analytical Technology and Instrumentation, School of Life Science and Technology, Xi’an Jiaotong University, Xi’an, Shaanxi China; 7 Department of Medicine, Geisel School of Medicine, Dartmouth College, Hanover, NH USA; 8 CAS Key Laboratory of Molecular Imaging, Beijing Key Laboratory of Molecular Imaging, The State Key Laboratory of Management and Control for Complex Systems, Institute of Automation, Chinese Academy of Sciences, Beijing, China

**Keywords:** Cancer imaging, Preclinical research, Biomedical engineering

## Abstract

Hypoxia in solid tumors is thought to be an important factor in resistance to therapy, but the extreme microscopic heterogeneity of the partial pressures of oxygen (pO_2_) between the capillaries makes it difficult to characterize the scope of this phenomenon without invasive sampling of oxygen distributions throughout the tissue. Here we develop a non-invasive method to track spatial oxygen distributions in tumors during fractionated radiotherapy, using oxygen-dependent quenching of phosphorescence, oxygen probe Oxyphor PtG4 and the radiotherapy-induced Cherenkov light to excite and image the phosphorescence lifetimes within the tissue. Mice bearing MDA-MB-231 breast cancer and FaDu head neck cancer xenografts show different pO_2_ responses during each of the 5 fractions (5 Gy per fraction), delivered from a clinical linear accelerator. This study demonstrates subsurface in vivo mapping of tumor pO_2_ distributions with submillimeter spatial resolution, thus providing a methodology to track response of tumors to fractionated radiotherapy.

## Abstract (web summary)

Hypoxia in solid tumors is an important factor in radiation therapy resistance, but partial pressures of oxygen in the tissue are heterogenous and difficult to measure. Here the authors develop Cherenkov excited phosphorescence imaging using oxygen probe Oxyphor PtG4 to spatially track oxygen distributions in tumors during fractionated radiotherapy.

## Introduction

Low levels of partial pressure of oxygen (pO_2_) in tissue, commonly referred to as hypoxia, are known to reduce the potential for therapeutic radiation damage, inducing tumor resistance and facilitating tumor cells escape from treatment^1–3^. Meta-analyses reveal that improvements in regional control of cancer are primarily observed in patients with well-oxygenated tumors and not in patients with hypoxic tumors^4,5,6^. Attempts have been made in the past in clinical trials to reduce hypoxia by administration of carbogen and nicotinamide^7^ or to provide radiation boost in the hypoxic areas^8^, although no methodology has yet been widely adopted in clinics. A part of the problem is that the progress in understanding and clinically addressing consequences of hypoxia has been limited by the lack of methods for non-invasive, fast, and repeatable mapping of pO_2_ distributions in tumor tissues. Multiple-track oxygen electrode measurements^9^ or ex vivo tissue immunohistochemistry^10^ suffer from invasiveness, which limits their clinical utility. Macroscopic assessment of tumor oxygenation in humans has been performed by positron emission tomography (PET) with fluoromisonidazole (FMISO) hypoxia tracer^11,12^ and by blood oxygen level dependent magnetic resonance imaging (BOLD MRI)^13,14^. However, these macroscopic imaging modalities are not able to fully sample the heterogeneity of the tumor microenvironment, while both chronic and transient hypoxia are documented to be microregional^15^. Additionally, measurements by BOLD MRI report on changes in the vascular pO_2_, whereas in tissues with abnormally developed vasculature, such as tumors, blood oxygen levels may be only poorly correlated with extravascular “tissue” pO_2_, and tissue oxygen may be misrepresented. Hence, measurements of intratumoral pO_2_ between capillaries and assessment of the heterogeneity of the pO_2_ distributions were the key motivating factors for this study.

Oxygen-dependent quenching of phosphorescence^16,17^ has been used in the past to map oxygen distributions in subcutaneous tumors in animal models^18–21^ both macroscopically and microscopically, depending upon the magnification of the imaging method used. These measurements have revealed correlations between tumor pO_2_ levels and radiation sensitivity^22,23^. However, truly high-resolution depth-resolved imaging by phosphorescence is possible only at relatively shallow depths, not exceeding 0.5–0.7 mm, by means of two-photon microscopy^24–27^, while volumetric phosphorescence lifetime tomography^28^ suffers from low resolution similar to other diffuse tomographic methods.

Recently, a new approach has been developed that combines phosphorescence quenching with excitation by the Cherenkov light generated within tissues that are subjected to high-energy radiation during fractionated radiotherapy^29–31^. This method, termed Cherenkov excited luminescence imaging (CELI), is used in the present study with the goal to directly image tumor pO_2_ distributions precisely at the time of radiation delivery. It is well known that hypoxic changes can cycle on the timescale of minutes, which is the typical time of a single radiation dose fraction^32–34^. Thus, the ability to image intratumoral pO_2_ at the actual time of the radiation delivery can be highly informative for optimization of clinical radiation therapy. In broad-beam CELI luminescence can be imaged that originates from the axial depths of up to 3–6 mm within the tumor, while the lateral spatial resolution may be as high as several hundred microns, depending on the luminescent source depth^31^. These features make CELI intrinsically capable of much higher depth resolution than all-optical tomographic imaging methods.

In this study, time-resolved CELI in combination with the phosphorescent probe Oxyphor PtG4 (Supplementary Fig. 1) is used to track oxygen changes in mouse xenograft tumors. Oxyphor PtG4 belongs to the family of dendritic oxygen probes^21,35^. Its calibration parameters are stable in biological environments, and its phosphorescence lifetime reports selectively on absolute pO_2_ levels in tissues. Cherenkov light generated within the tumors, subjected to pulsed therapeutic megavolt (MV) X-ray irradiation, served as an internal light source to excite the probe’s phosphorescence. A time-gated intensified charge coupled device (ICCD) camera, synchronized with the excitation pulses, captures emission at different delay times after each pulse (Supplementary Fig. 2a). The images of phosphorescence at different delays are used to construct an image of phosphorescence lifetimes by applying pixel-wise exponential fitting (Supplementary Fig. 2b). Notably, the tissue pO_2_ values are obtained from the phosphorescence lifetimes, as opposed to intensities, therefore being unaffected by optical heterogeneities of the medium and insensitive to the non-uniformity of the probe distribution. The practical usefulness of our imaging approach is evaluated by combining it with the hypofractioned radiation therapy scheme, whereby a single intravascular injection of Oxyphor PtG4 allows us to track oxygen dynamics in tumors during the course of the entire treatment.

## Results

### Bio-distribution of Oxyphor PtG4

**Fig. 1:**
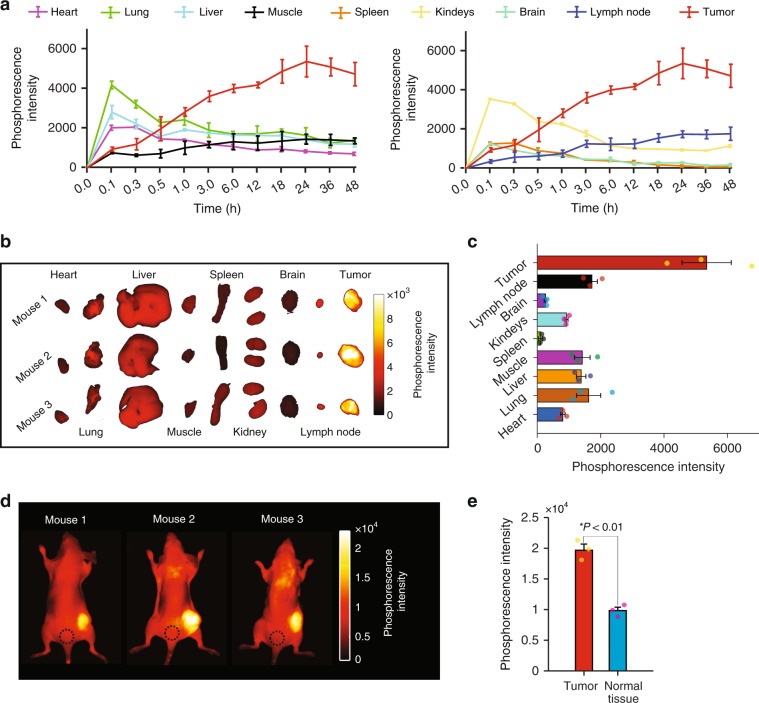
Bio-distribution of Oxyphor PtG4 measured with IVIS instrument at different time points. **a** Phosphorescence from different organs and tumors (*n* = 3) measured at 12 time points after IV injection of Oxyphor PtG4 (*n* = 3 mice per time point, *n* = 36 mice total). Ex vivo images of the phosphorescence from the excised organs (**b**) and their integrated intensities (**c**) at the 24 h time point after IV injection of Oxyphor PtG4 (*n* = 3). In vivo images of the phosphorescence (**d**) and the phosphorescence intensities in the tumors and regions of the normal tissue (marked by a black-dashed circle) (**e**) at 24 h after injection of Oxyphor PtG4. The mean signal from the tumors (1.97 ± 0.16 × 10^4^ counts, mean ± SE) was twice as high as that from the normal tissue (0.98 ± 0.09 × 10^4^ counts, mean ± SE) (*n* = 3). Error bars represent standard error of the mean. Significant difference was analyzed by two-sample *t*-test.

To evaluate the bio-distribution of Oxyphor PtG4 its phosphorescence was imaged ex vivo at different time points using a standard wide-field optical imaging system (IVIS 200 Spectrum CT, Perkin-Elmer, USA) after intravenous (IV) injection into the tail vein. The phosphorescence of Oxyphor PtG4 reached its peak within 5 min in the heart, lung, liver, spleen, kidneys and brain after the injection, while the highest signal in the tumor was detected after 24 h (Fig. 1a). The ex vivo images of the phosphorescence in individual organs at the 24 h time point are shown in Fig. 1b along with the quantitative image analysis (Fig. 1c). Consistent with the ex vivo results, the images obtained in vivo (Fig. 1d), also at the 24 h time point, show that the phosphorescence from the tumors is ~2 times brighter than that from the normal tissue (Fig. 1e).

### Long-term retention of Oxyphor PtG4 in tumors

**Fig. 2:**
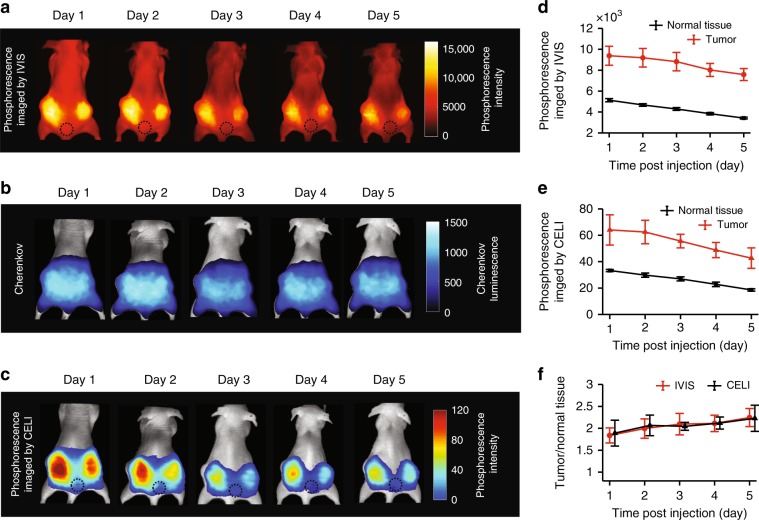
Longitudinal in vivo imaging of phosphorescence during 5 days after a single IV injection of Oxyphor PtG4. **a** Phosphorescence intensity images measured using wide-field optical excitation in a standard IVIS instrument. **b** Integrated intensity images of the Cherenkov light along with the phosphorescence of Oxyphor PtG4, acquired during the time of the radiation delivery. **c** Phosphorescence intensity images by CELI. Average intensity of the phosphorescence acquired by IVIS (**d**) and CELI (**e**) for the tumor area and for the normal tissue (*n* = 4). **f** The ratio of the signals tumor:normal tissue as imaged by IVIS and CELI (*n* = 4). Error bars represent standard error of the mean.

Four mice were imaged using the IVIS instrument as well as by CELI every day for 5 days after IV injection of Oxyphor PtG4. The radiation for CELI was delivered using a beam of a large diameter, such that the images encompassed the entire abdomen area, including both tumors, as shown in the images of the excitation Cherenkov light (Fig. 2b). The phosphorescence images acquired using either IVIS or CELI indicated that sufficient amounts of Oxyphor PtG4 lasted in the tumor for at least 5 days, which provided an opportunity to image oxygenation in these tumors during multiple-fraction radiotherapy upon a single Oxyphor PtG4 injection (Fig. 2a, c). The phosphorescence intensity of Oxyphor PtG4 in the tumor and the normal tissue decreased monotonously from day 1 to day 5 (Fig. 2d, e). Since the signal intensity both in the normal tissue and tumor decreased gradually during the 5 days, the ratio “tumor signal:normal tissue signal” showed no significant decline from day 1 to day 5 (Fig. 2f). Notably, the overall intensity of the signals measured using optical excitation was much higher than that in CELI, because the intensity of the light used for excitation in the IVIS  instrument is much higher than that of the Cherenkov light. However, the Cherenkov radiation is generated within the tissue, and therefore the excitation has virtually no depth limitation, while the external optical excitation attenuates exponentially with depth (Supplementary Fig. 3a, b). As a result, CELI is capable of sampling oxygenation in much deeper tissue layers than all-optical phosphorescence imaging (Supplementary Fig. 3c, d).

### In vivo pO_2_ imaging after local injection of Oxyphor PtG4

**Fig. 3:**
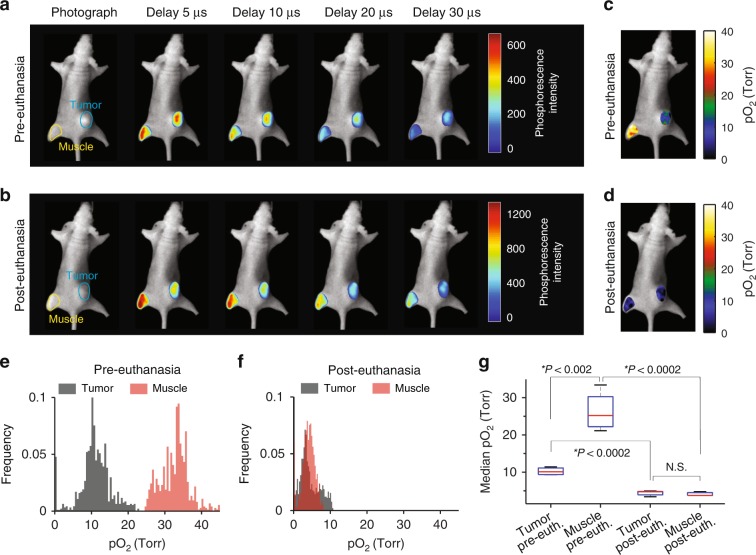
CELI of oxygen in tumors and muscle in vivo before and 30 min after euthanasia. Oxyphor PtG4 (50 μL of 200 μM solution) was locally injected into the MDA-MB-231 tumor (blue circle) and into muscle (yellow circle) before the imaging session. **a**, **b** Phosphorescence intensity images acquired at different delays relative to the radiation pulse before (**a**) and after euthanasia (**b**). The phosphorescence images are overlaid on a photograph of the mouse, which was taken before CELI. The tumor and muscle areas used for the analysis are encircled. Tissue oxygen maps (**c**, **d**) and pO_2_ histograms (**e**, **f**) before and after euthanasia. **g** Average levels of oxygen in the tumor and muscle before and after euthanasia (*n* = 4). Boxplot shows median and interquartile range; whiskers indicate the range. Statistics was performed using two-sample *t*-test.

To demonstrate the ability to image pO_2_ in vivo by CELI in healthy (muscle) and tumor tissues pre- and post-euthanasia, Oxyphor PtG4 was locally injected into the tumor and into a thigh muscle. CELI was performed in four mice, first before (Fig. 3a) and then 30 min after (Fig. 3b) euthanasia. The data processing followed the standard protocol for wide-field phosphorescence lifetime imaging^21^. The accuracy of the lifetime measurements by CELI was verified independently by performing ex vivo measurements using standard lifetime measurement instrumentation (Supplementary Fig. 4). The pO_2_ images (Fig. 3c, d) were calculated from the lifetime images using the Stern–Volmer equation and the calibration constants obtained in independent measurements. In live animals the pO_2_ values in the tumors were significantly lower than in the muscle tissue (Fig. 3c, e). As expected, the pO_2_ values both in the tumors and muscle rapidly dropped to near zero when mice were euthanatized (Fig. 3d, f). There was a significant variation between the median pO_2_ values for the tumor and muscle tissues in the four mice before euthanasia, but after euthanasia the pO_2_ was uniformly near zero in all animals (Fig. 3g).

### In vivo pO_2_ imaging after IV injection of Oxyphor PtG4

**Fig. 4:**
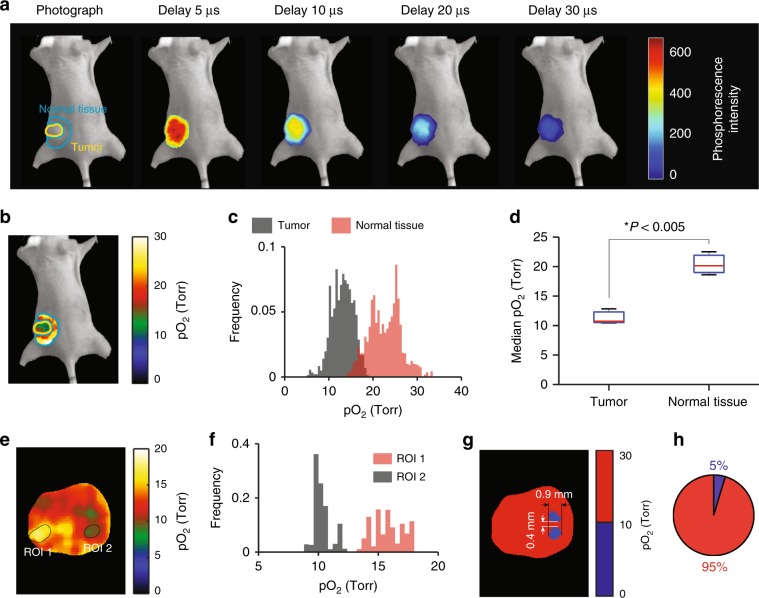
In vivo imaging of pO_2_ in mice 24 h after IV injection of Oxyphor PtG4. **a** Phosphorescence intensity images acquired at different delays of 5, 10, 20, and 30 µs relative to the excitation pulse, overlaid with a photograph of the mouse. The regions corresponding to the tumor and the normal surrounding tissue are shown by the yellow and blue circles in the first image. **b** Tissue pO_2_ map. **c** pO_2_ histograms. **d** Median pO_2_ values in the tumor and surrounding tissue (*n* = 3). Boxplot shows median and interquartile range; whiskers indicate the range. Statistics was performed using two-sample *t*-test. **e** Enlarged view of the tumor region in the pO_2_ image. **f** pO_2_ histograms of sub-regions (ROIs) outlined by black curves in **e**. **g** Areas shown in blue were characterized by pO_2_ < 10 Torr. **h** Fraction of the tumor having pO_2_ < 10 Torr.

Three mice were IV injected with Oxyphor PtG4 (200 μL of 200 μM solution) through the tail vein, so that the final concentration of the probe in the blood was ~20 µM, and CELI was performed 24 h later (Fig. 4a). The pO_2_ images (Fig. 4b) and their histograms (Fig. 4c) revealed a significant degree of hypoxia in the tumors versus the surrounding tissue. Statistically significant differences (*P* < 0.005, *n* = 3, two-sample *t*-test) were observed between the median pO_2_ values (Fig. 4d) in the tumor (11.3 ± 0.8 Torr, mean ± SE, *n* = 3) and normal tissue (20.4 ± 1.1 Torr, mean ± SE, *n* = 3). The pO_2_ distributions in the tumor regions were found to be highly heterogeneous (Fig. 4e). Analysis of locally high and low pO_2_ sub-regions revealed that they are characterized by significantly different pO_2_ histograms (Fig. 4f). An arbitrary threshold of 10 Torr was applied to divide the pO_2_ image into hypoxic (pO_2_ < 10 Torr) and normoxic (pO_2_ ≥ 10 Torr) regions. According to this division, there were two hypoxic regions in the tumor (Fig. 4g), which accounted for 5% of the imaged area (Fig. 4h). These regions were smaller than 1 mm in diameter and were separated by a distance of 0.4 mm (Fig. 4e).

### In vivo pO_2_ imaging during multiple-fraction radiotherapy

**Fig. 5:**
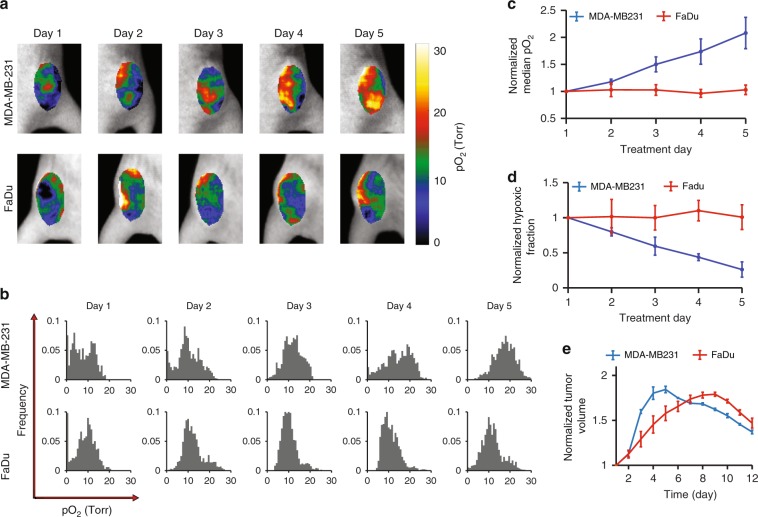
In vivo longitudinal pO_2_ imaging of mice with two tumor lines during 5 days-long fractionated radiotherapy. **a**, **b** Examples of pO_2_ images (**a**) and of histograms (**b**) acquired during each day of the radiotherapy treatment. **c**, **d** Median pO_2_ changes (**c**) and hypoxic fraction changes (**d**). Hypoxic fraction is defined here as the ratio of the area with pO_2_ < 10 Torr to the total tumor area. The changes in the median pO_2_ (**c**) and in the hypoxic fraction (**d**) are shown relative to the respective values on the first treatment day. **e** Relative tumor volume changes during and after the radiotherapy, normalized by the volume prior to the treatment. All data are shown as mean ± standard error of the mean (*n* = 3 in **c**, **d**, **e**).

Six mice with subcutaneous MDA-MB-231 or FaDu tumors were imaged while undergoing 5 days-long multiple-fraction radiotherapy. The radiation was delivered using a 6MV X-ray beam with 5 Gy/fraction daily, with the total radiation dose of 25 Gy. Oxyphor PtG4 was IV injected 24 h before the first treatment, and CELI was performed during each fraction of radiation. The pO_2_ distributions in both MDA-MB-231 and FaDu tumors revealed a high degree of heterogeneity^36,37^. Importantly, the hypoxic regions decreased in size from one fraction to the next, and the decrease was much more pronounced in the case of the MDA-MB-231 tumors (Fig. 5a). In both tumor types, the pO_2_ values were below 20 Torr on the first day, and they increased up to ~30 Torr for MDA-MB-231 and up 25 Torr for FaDu tumors (Fig. 5b). The median pO_2_ values in the MDA-MB-231 tumors increased markedly as the radiotherapy progressed, while no obvious changes were seen in the case of FaDu tumors (Fig. 5c), implying that more hypoxic and radio-resistant tumors allowed for less local control, which presumably resulted in a higher rate of their survival^38^ (Fig. 5d). The response of the tumor volume was delayed relative to the response of the local pO_2_, i.e. MDA-MB-231 tumors started to decrease in size on day 5 and FaDu tumors only on day 9 from the start of the therapy (Fig. 5e). Overall, the changes in the pO_2_ distributions and the tumor volumes suggest that MDA-MB-231 tumors are more prone to the radiation damage compared to FaDu tumors (see Supplementary Fig. 5 for additional results and Supplementary Fig. 6 for histological evaluation of the tumor tissue after radiation).

## Discussion

There is a need to measure pO_2_ distributions in tumors with high spatial resolution^39–41^ in order to identify hypoxic fractions, which are known to be less sensitive to radiation. CELI in combination with Oxyphor PtG4 may help to identify such hypoxic regions and thus help navigating and optimizing clinical radiation therapy. The fact that CELI can be performed simultaneously with fractionated therapy makes it particularly clinically relevant. In this version of CELI the limiting factor is the optical perspective of view, i.e. clear optical access to the tumor is required. In addition, the method is capable of sensing pO_2_ only in a few millimeters thick layer of subcutaneous tissue. However, future realizations of the technique could be adapted for imaging of intracavitary or near-surface tumors. Most importantly, while this imaging method presently can be utilized only in pre-clinical studies, it is uniquely capable of non-invasive sampling of pO_2_ across tumors with submillimeter spatial resolution exactly at the time of the radiotherapy delivery.

In this work we showed that Oxyphor PtG4 accumulates preferentially within tumors and remains there for at least 5 days. This localization is likely due to the enhanced permeability and retention (EPR) effect. Localization studies (Supplementary Fig. 7) revealed that the probe distributes across the entire interstitial space of the tumor. There may be slightly higher sequestration into necrotic areas at later time points, but there is also more necrosis at these time points, making this further sequestration pattern hard to assay with certainty. Importantly, the probe is present in the tumors throughout the entire time course of the studies, and stronger quenching of the phosphorescence at higher oxygen concentrations, characteristic of normal tissues, results in the phosphorescence from hypoxic tumors being significantly brighter (Fig. 1e), providing a natural signal selection mechanism.

A single injection of Oxyphor PtG4 allowed pO_2_ imaging over multiple days during fractionated radiotherapy. Since pO_2_ is estimated from the phosphorescence lifetime, the decrease in the concentration of the probe due to its clearance from the body does not affect the ability to quantify oxygen, as long as enough probe is present for acquiring adequate signals. Longer-term residence of Oxyphor PtG4 remains to be investigated as well as potential long-term toxicity issues; however, cell metabolic activity measurements using a standard MTT assay showed that the cell viability was above 90% when the cells were incubated with Oxyphor PtG4 at the concentration of 40 µM for 24 h (Supplementary Fig. 8), that is two times higher than in all in vivo experiments performed in this study. A similar result has been obtained previously in the toxicity tests of the probe Oxyphor PdG_4_^42^, which is structurally almost identical to Oxyphor PtG4. Hematoxylin and eosin staining assay did not show differences between tissues of the main organs of animals infused with Oxyphor PtG4 vs phosphate buffer saline (PBS) (Supplementary Fig. 9). Likewise, blood chemistry tests (Supplementary Table S1) revealed no difference between treatments with Oxyphor PtG4 vs PBS. Overall, our results suggest that Oxyphor PtG4 has no toxic effects on the physiological function of cells, and its clearance from the body occurs presumably by the renal process.

The excitation source in CELI is the Cherenkov light generated along the pathways of MV X-ray beams. Therefore, excitation occurs at all depths where the radiation dose is deposited, including deep within tissues. Nevertheless, the detection of the phosphorescence emission is still limited by the tissue absorption and scattering. Considering the limitations of imaging sensors and the moderate brightness of the partially quenched phosphorescence, the maximal imaging depth is currently estimated to be ~2 cm (Supplementary Fig. 3). The depth can be increased by employing brighter phosphorescent probes, such as recently developed Oxyphor 2P^43^ and/or using cameras with sensitivity optimized specifically for the phosphorescence spectral range (750–850 nm). The scope of potential applications of CELI then can be extended on imaging of near-surface tumors, such as head and neck tumors, as well as on pre-clinical assessments of oxygenation during therapy in laboratory animals. It is also possible that intracavity measurements could be achieved with appropriate catheter-based cameras.

This study was specifically designed to use clinically relevant stereotactic hypofractionated radiotherapy doses and 6MV X-rays from a clinical LINAC system. In our recent work we demonstrated the possibility of high-resolution CELI during standard clinical intensity-modulated radiotherapy in a whole-breast geometry. We do not anticipate technical difficulties in adapting the latter method for fractionated treatment^44^.

Compared to the current clinical pO_2_ measurement modalities, the CELI approach is capable of significantly higher spatial resolution and allows image acquisition simultaneously with radiotherapy delivered in multiple fractions. One current limitation of the technique is that only two-dimensional pO_2_ images can be obtained. Our previous studies have shown that three-dimensional (3D) tomographic imaging of phosphorescence using light sheet excitation and diffuse optical tomography image reconstruction methods makes it possible to obtain 3D pO_2_ maps with submillimeter resolution in vivo^29^. However, the tomography technique relies on thin sheet-like X-ray beams, which are not routinely used in the current clinical practice. Furthermore, scanning of the entire tissue volume for tomographic reconstructions may be difficult to incorporate into protocols of clinical therapy. In contrast, the simplified method reported here can be easily added to clinical protocols, enabling evaluation of tumor pO_2_ histograms at the time of the radiation dose delivery, which is a long-sought goal in tumor therapy.

## Methods

### Ethics statement

Experimental procedures involving live animals were carried out in accordance with the protocols approved by Dartmouth Institutional Animal Care and Use Committee (Protocol Numbers 00002173). Subcutaneous and intravenous injections were performed under anesthesia that was induced and maintained with isoflurane. All efforts were made to minimize animal suffering.

### Oxygen probe Oxyphor PtG4

Oxyphor PtG4 belongs to the family of dendritic oxygen probes^35^. It is a direct analog of the previously published probe Oxyphor PdG_4_^21^, different only by the metal in the porphyrin: platinum vs palladium (Supplementary Fig. 1a). The porphyrin is responsible for the key optical properties of the probe, which include two intense absorption bands (*λ*_max_ = 435 and 624 nm) and strong phosphorescence (*λ*_max_ = 780 nm, QY~0.07 in deoxygenated aq. solutions) occurring in the tissue near-infrared window (Supplementary Fig. 1b). The porphyrin molecule is encapsulated in a hydrophobic jacket, formed by a folded aryl-glycine dendrimer. The dendrimer protects the porphyrin from interactions with endogenous macromolecules and attenuates the rate of oxygen diffusion in the vicinity of the porphyrin, bringing the probe’s sensitivity into the desired pO_2_ range. The exterior of the probe is modified with multiple polyethyleneglycol (PEG) residues, making it hydrophilic and inert with respect to the endogenous macromolecules. Due to the extensive PEGylation the probe’s calibration parameters remain unaltered in biological environments. The molecular weight of Oxyphor PtG4 is ~35 kDa, and the approximate diameter of the molecule in an aqueous solution is ~6 nm. Therefore, Oxyphor PtG4 can easily diffuse into the tumor interstitial space from the leaky vasculature: a phenomenon known as EPR effect. However, the same PEG coating prevents Oxyphor PtG4 from permeating cellular membranes. Thus, measurements using Oxyphor PtG4 give average intratumoral extravascular pO_2_.

The phosphorescence lifetime of Oxyphor PtG4 at 37 °C under fully anoxic conditions is *τ*_0_ = 45 μs. The response of the phosphorescence to oxygen was calibrated by simultaneously measuring the probe’s phosphorescence lifetime and pO_2_ in solution using a high-precision oxygen electrode, while gradually decreasing oxygen concentration by substituting oxygen with argon^21^. The Stern–Volmer oxygen quenching plot was linear throughout the physiological pO_2_ range (0–150 Torr) with the quenching constant *k*_q_ = 366 Torr^−1^ s^−1^. In addition, the probe was calibrated using Cherenkov excitation by LINAC pulses and four acquisition windows with delays of 5, 10, 20, and 30 μs. The signal integration time after each delay was 200 μs, and 360 windows were accumulated for each delay. This latter calibration was performed using an air-equilibrated solution of Oxyphor PtG4 (pO_2_ = 150 Torr at 37 °C) and a fully deoxygenated solution (pO_2_ = 0 Torr at 37 °C) (Supplementary Fig. 1c).

### Tumor cell lines

The human breast cancer cell line MDA-MB-231 was purchased from Perkin-Elmer/Caliper (Cat. No.: 119369), and human head neck cancer cell line FaDu was purchased directly from American Type Culture Collection (ATCC HTB-43, Manassas Virginia). These are not listed in the ICLAC database of cross-contaminated or misidentified cell lines. Cells were grown in culture media in a humidified incubator at 37 °C and 5% CO_2_ in MEM with 10% (v/v) fetal bovine serum (FBS), 100 U/mL penicillin, and 100 μg/mL streptomycin. When ready for use, the cells were trypsinized, counted, pelleted, and resuspended for injection.

### Animal preparation

All procedures followed the protocol approved by the Dartmouth Institutional Animal Care and Use Committee. Nude female mice 6–8 weeks of age (Charles River Labs, Wilmington, MA) were involved in this study. The mice were housed in the Dartmouth central animal facility and fed special diet—MP biomedical purified diet. 10^6^ MDA-MB-231 or 10^6^ FaDu tumor cells were injected subcutaneously under the skin on the flank of each mouse. After approximately 2 weeks of growth, animals were chosen for imaging when their tumor diameter reached approximately 8 mm in size. On the day of the initial use, mice were anesthetized, and Oxyphor PtG4 was either IV injected into a tail vein (200 μL of 200 μM solution) or locally injected into the tumor or normal muscle (50 μL of 200 μM solution), right before the start of the first in vivo imaging session.

### In vitro imaging of bio-distributions of Oxyphor PtG4

Wide-field optical imaging (IVIS 200 Spectrum CT; Perkin-Elmer, USA) was used to quantify phosphorescence from harvested organs (heart, lung, liver, spleen, kidneys, brain, muscle, lymph node, and tumor). The excitation and emission wavelengths were 640 and 780 nm, respectively. The integration time was 0.2 s with binning of 2. In total, 36 mice with MDA-MB-231 tumors were IV injected with Oxyphor PtG4 (100 μL of 100 μM solution) through the tail vein. At each of the 12 time points of 0 min, 5 min, 15 min, 30 min, 1 h, 3 h, 6 h, 12 h, 18 h, 24 h, 36 h, and 48 h, organs from three mice were imaged.

### CELI during radiotherapy

Cherenkov light was induced in the tissue by irradiation from a linear accelerator (Varian Linac 2100CD; Varian Medical System) at the Norris Cotton Cancer Center, Dartmouth-Hitchcock Medical Center. The beam size was adjusted to cover the entire tumor area. To minimize the background, all lights in the room were switched off. The imaging system consisted of a time-gated intensified CCD camera (ICCD, PI-MAX4 1024i; Princeton Instruments), a lens (Canon EF 135 mm f/2L USM), and a 750 nm/100 nm band pass filter. The camera was contained in a homemade lead box, placed ~2 m away from the imaging field to protect it from Compton scattered X-ray photons. The noise induced by X-ray photons was therefore significantly suppressed, and the signal-to-noise ratio (SNR) was more than seven times higher after shielding (Supplementary Fig. 10). The time-gated ICCD camera was synchronized with the radiation pulses, which were ~3.25 μs long and delivered with 360 Hz repetition rate. The camera gate was turned on at 5, 10, 20, and 30 μs delay times and the phosphorescence was integrated on the CCD for 200 μs. The intensifier gain was ×100.

### Longitudinal imaging study

The mice injected with Oxyphor PtG4 were continuously imaged by CELI for 5 days during their hypofractionated radiotherapy, and also imaged with the IVIS instrument for comparative purposes. All mice were under general anesthesia (isofluorane 1–3% admixed to O_2_ and delivered through a nose cone) throughout imaging.

### Cytotoxicity assay

MTT assays were performed on MDA-MB-231 and Fadu cells. Solutions of Oxyphor PtG4 at different concentrations (0, 10, 20, and 40 μM diluted in PBS) were added to the wells. The samples were incubated for 24 h in a cell culture incubator, and 10 μL of 5 mg/mL MTT solution [3-(4,5-dimethylthiazolyl-2)-2,5-diphenyl tetrazolium bromide; MP Biomedical, Solon OH] was added to each well. After incubating the plate for another 6 h, the media was removed from all wells and 150 μL of acidified isopropanol was added to each well. Finally, the percentage of living cells was determined based on the measured optical density (OD) at 540 nm.

### Statistical analysis

All statistical analyses for differences in pO_2_ between treatment groups were done using two-sample *t*-test with Matlab (Mathworks, Inc., Natick, MA).

### Reporting summary

Further information on research design is available in the Nature Research Reporting Summary linked to this article.

## Supplementary information

Supplementary Information

Reporting Summary

## Data Availability

The data that support the findings of this study are available within the paper and its Supplementary Information. Source datasets generated and analyzed during the study are available from the corresponding author upon request.

## Code availability

MATLAB routines for this study are available from the corresponding author upon request.
