# Supplementary material for: Tissue pO2 distributions in xenograft tumors dynamically imaged by Cherenkov-excited phosphorescence during fractionated radiation therapy
Source: Nat Commun. 2020 Jan 29;11:573. doi: 10.1038/s41467-020-14415-9 (PMC6989492; doi:10.1038/s41467-020-14415-9)
Supplement: Supplementary file 1 — Supplementary Information [file 41467_2020_14415_MOESM1_ESM.pdf]

Supplementary Information

Tissue pO_2 Distributions in Xenograft Tumors Dynamically Imaged by Cherenkov-Excited Phosphorescence during Fractionated Radiation Therapy

Cao et al.

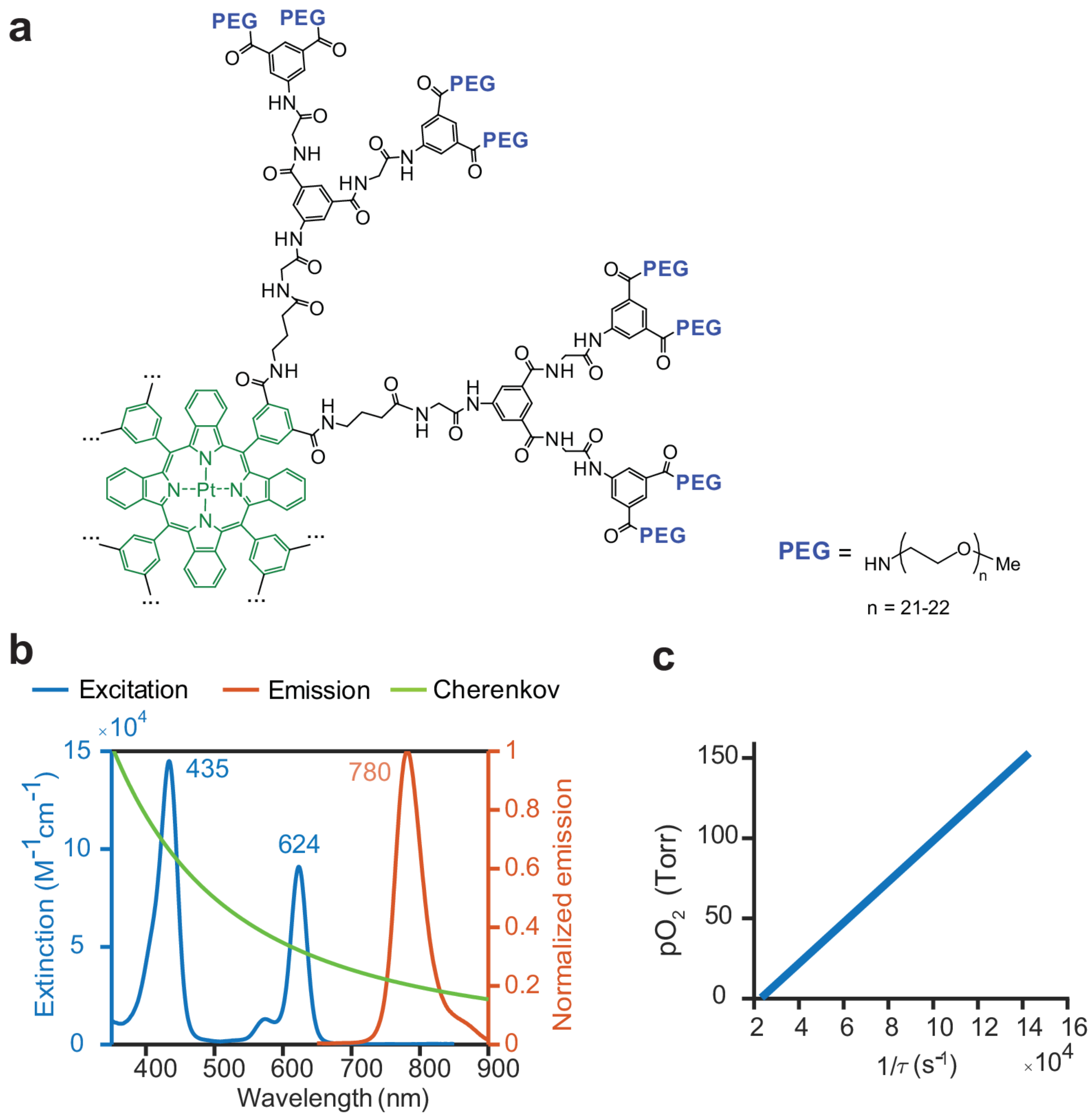

Supplementary Figure 1 Structure and Properties of Oxyphor PtG4. (a) Structure and optical spectra of Oxyphor PtG4. (b) Excitation and emission spectra of Oxyphor PtG4 and the spectrum of Cherenkov radiation. The absorption bands of Oxyphor PtG4 (λ_{max}=435 nm, ε~140,000 M^{-1} cm^{-1}, λ_{max}=624 nm, ε~90,000 M^{-1} cm^{-1}) strongly overlap with the Cherenkov spectrum, ensuring efficient excitation, while the phosphorescent photons (λ_{max}=780 nm) are minimally absorbed by the tissue and can easily diffuse from the depth and be detected by outside cameras. (c) Oxygen quenching plot of Oxyphor PtG4. The pO_2 values in solution of Oxyphor PtG4 are plotted as a function of the inverse of the phosphorescence lifetime.

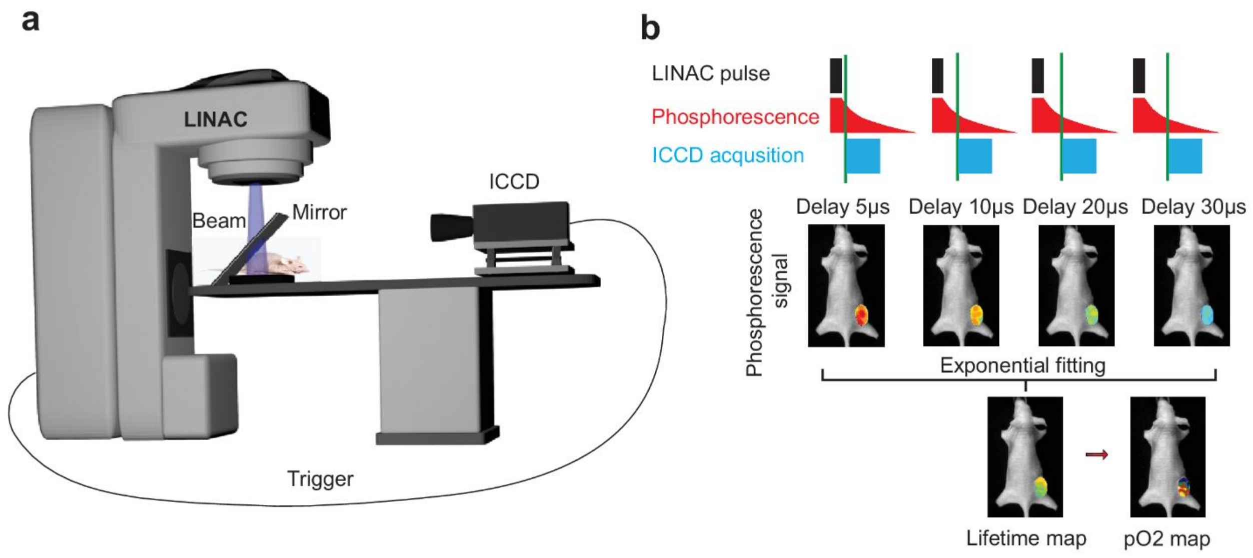

Supplementary Figure 2 pO_2 mapping by CELI. (a) Diagram of experimental setup. The 6MV photon beam was delivered to generate Cherenkov light and excite the probe's phosphorescence. An intensified charge-coupled device (ICCD) camera was trigged by synchronized pulse from Linac to acquire phosphorescence of the probe at different delay times. (b) Data acquisition and processing. Four CELI images were acquired with delay times of 5μs, 10μs, 20μs and 30μs. The phosphorescence lifetimes in each pixel were calculated by single exponential fitting, and the pO_2 values were calculated using the calibration parameters.

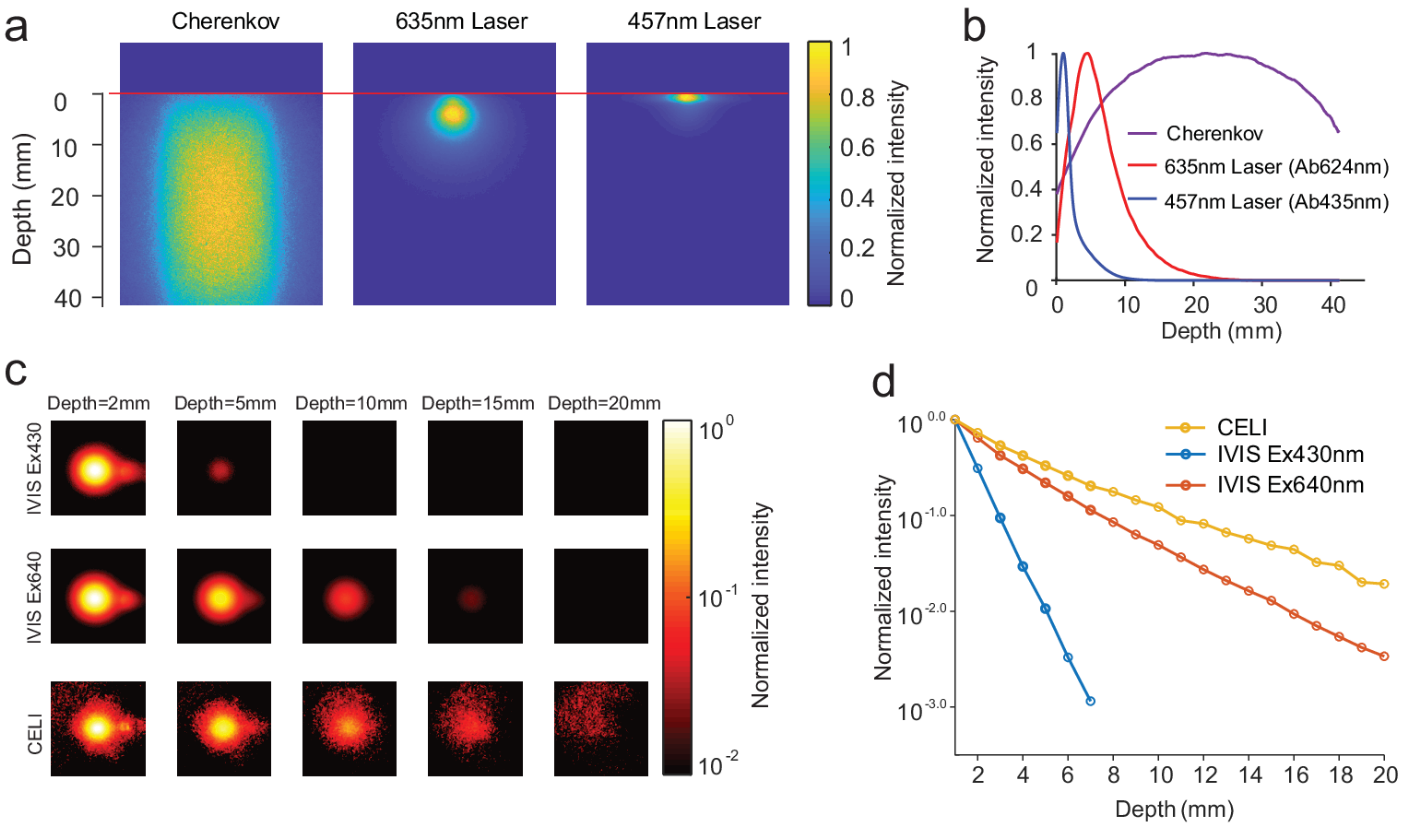

Supplementary Figure 3 Comparison between CELI and IVIS imaging. (a) Images of depth distributions of Cherenkov light, and the emission of 635 nm and 457 nm lasers in liquid medium (Phosphate buffer saline with 1% intralipid and 1% porcine blood). The lasers were chosen to approximate excitation of the two absorption peaks (624 nm and 435 nm) of Oxyphor PtG4. (b) Depth profiles calculated by adding pixels of all of the rows of the middle 20 columns in the images (a). (c) CELI and IVIS images of a tube filled with a solution of Oxyphor PtG4 immersed at different depths in the liquid medium. IVIS imaging was performed using excitation at either 430 nm or 640 nm - the wavelengths close to the absorption peaks of Oxyphor PtG4. (d) Quantitative analysis of the phosphorescence signals versus the depths in the liquid medium for CELI and IVIS imaging.

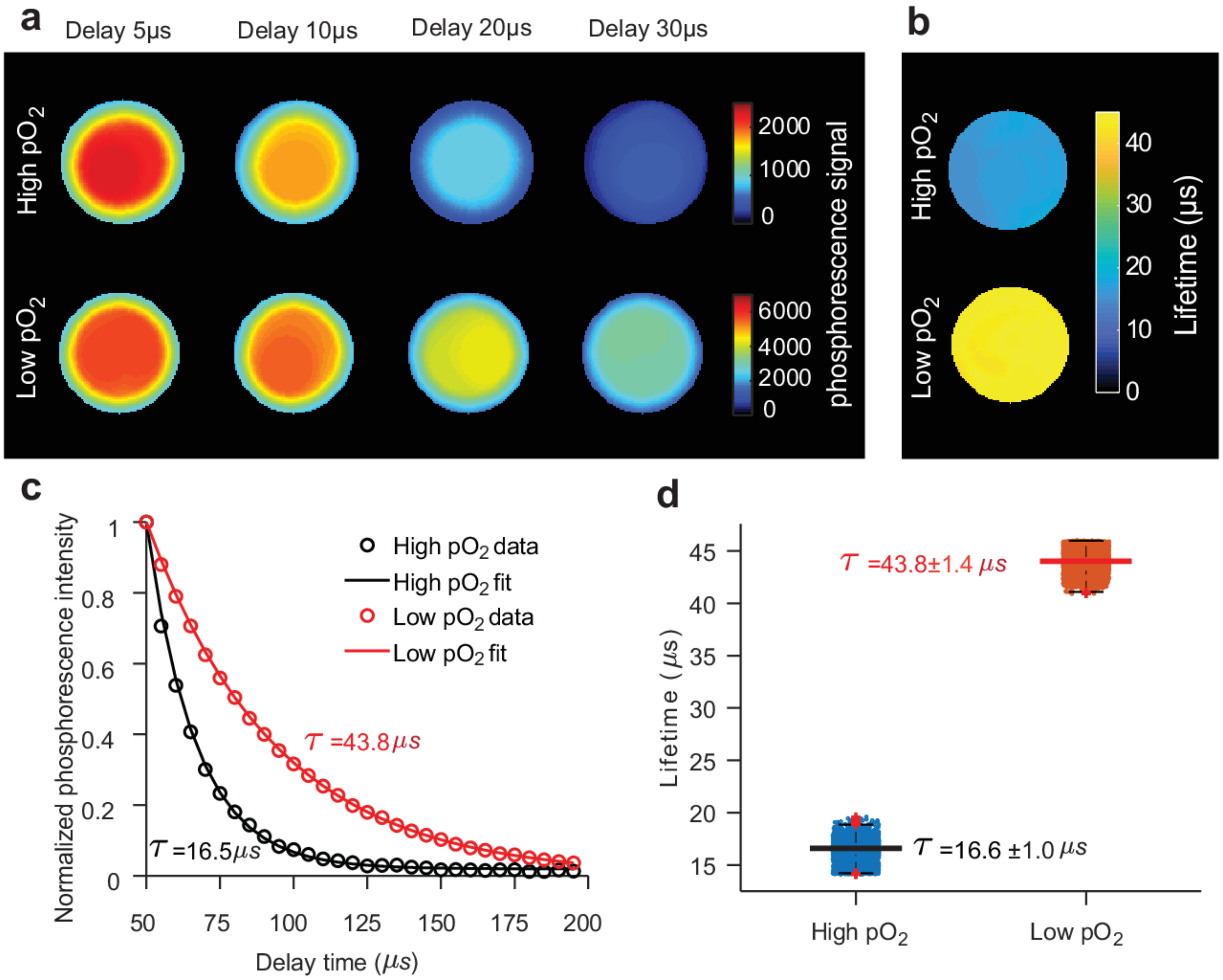

Supplementary Figure 4 Lifetime estimation based on CELI. (a) CELI images of the phosphorescence of Oxyphor PtG4 obtained at different delay times in low and high pO_2 environments. (b) The calculated lifetime map based on the CELI images. (c) Lifetime measurements using a standard fluorimeter with time-resolved measurement capabilities (FluoroMax4, Horiba Scientific, Japan) (d) Boxplot of lifetime values obtained by CELI.

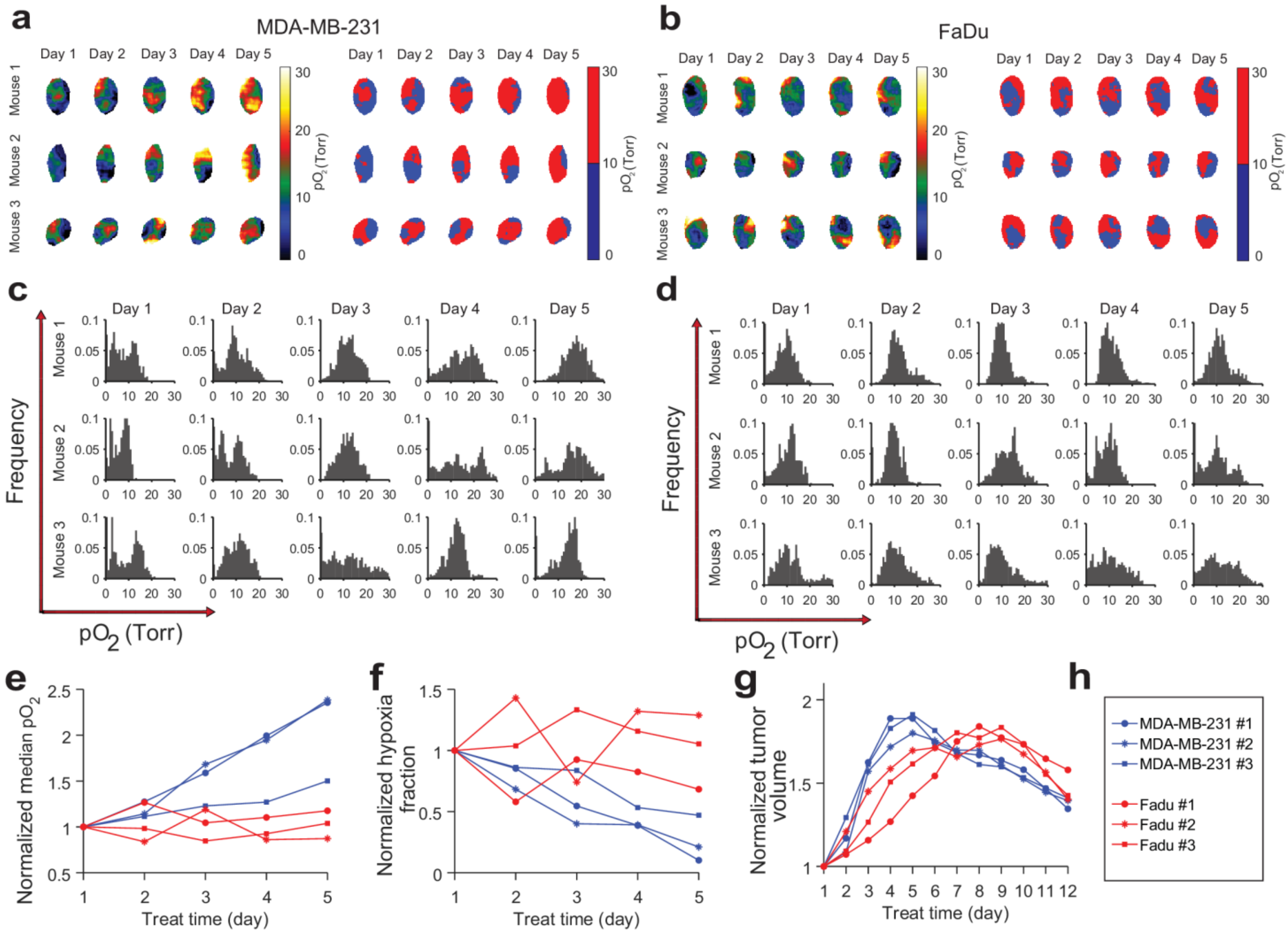

Supplementary Figure 5 In-vivo longitudinal pO_2 imaging of 6 mice (3 MDA-MB-231 and 3 FaDu tumors) during 5-days of fractionated radiotherapy. (a, b) pO_2 images and hypoxic region images of MDA-MB-231 tumor (a) and FaDu tumor (b). Hypoxic region images were defined by grouping the values in the pO_2 images using a threshold of 10 Torr; the areas shown in blue are designated as hypoxic, i.e. pO_2<10 Torr. (c, d) pO_2 histograms of a MDA-MB-231 tumor (a) and a FaDu tumor (b) measured during each day of the radiotherapy treatment. (e) Median pO_2 changes during the 5-days of radiotherapy relative to the start of the treatment. (f) Hypoxic fraction change defined by the proportion of pO_2<10 Torr during the 5-days of radiotherapy, normalized by the first measurement during the fraction treatment on day 1. (g) Fractional change in the tumor volume during and after the radiotherapy (normalized by the pre-treatment volume). (h) Key to all the curves shown in e, f, g.

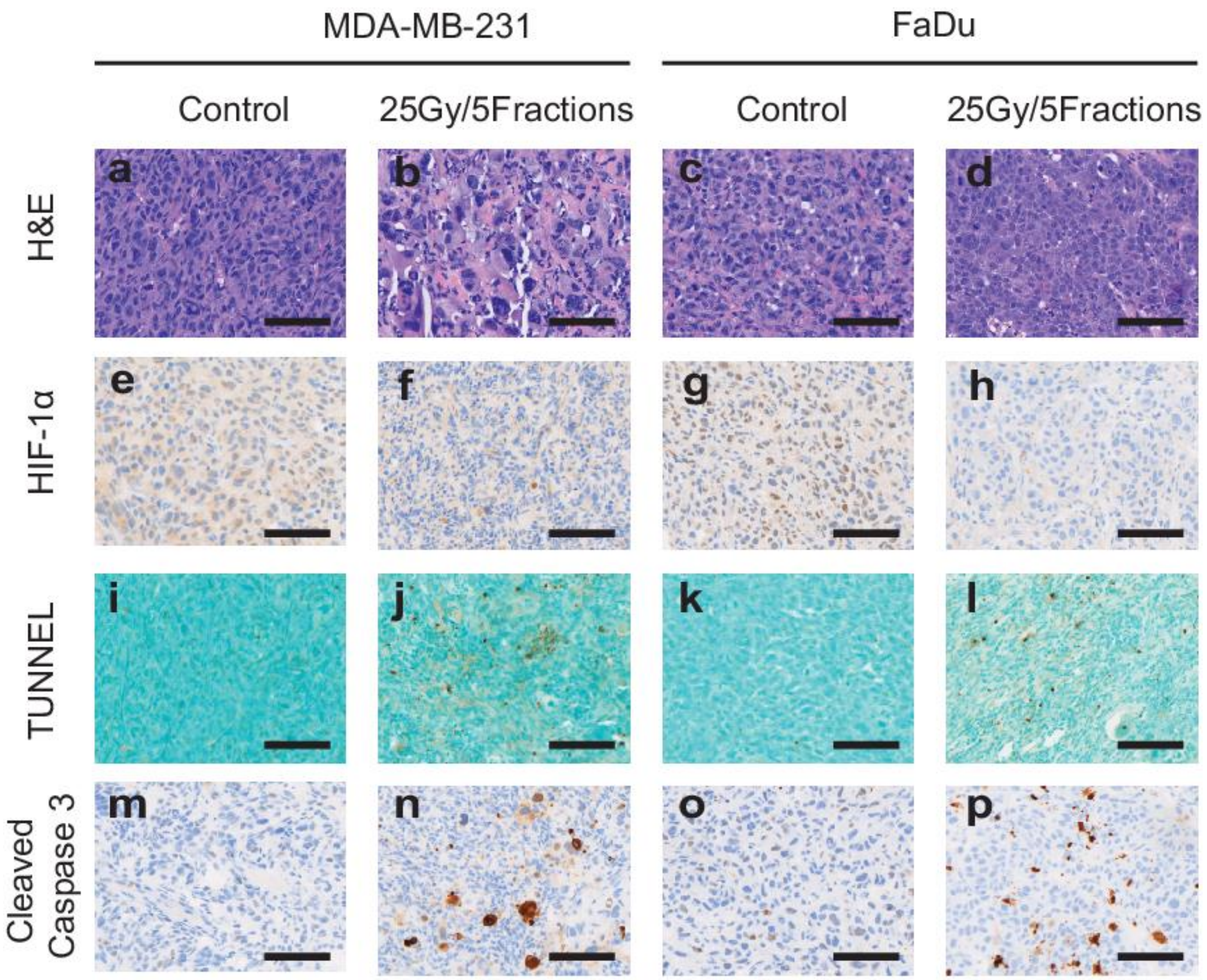

Supplementary Figure 6 Histological analyses of tumor sections response to radiotherapy. (a-d) Representative hematoxylin and eosin (H&E) stained histopathologic tumor sections. (e-h) Expression of hypoxia-inducible factor 1 alpha (HIF-1α) decreased in tumor cells when treated with radiotherapy compared to untreated tumors. (i-l) TUNEL staining of tumor sections. the brown stains indicate apoptotic cells. (m-p) Expression of cleaved caspase-3 activity in treated tumor sections. (scale bar: 100 μm; Control: the mice without radiotherapy).

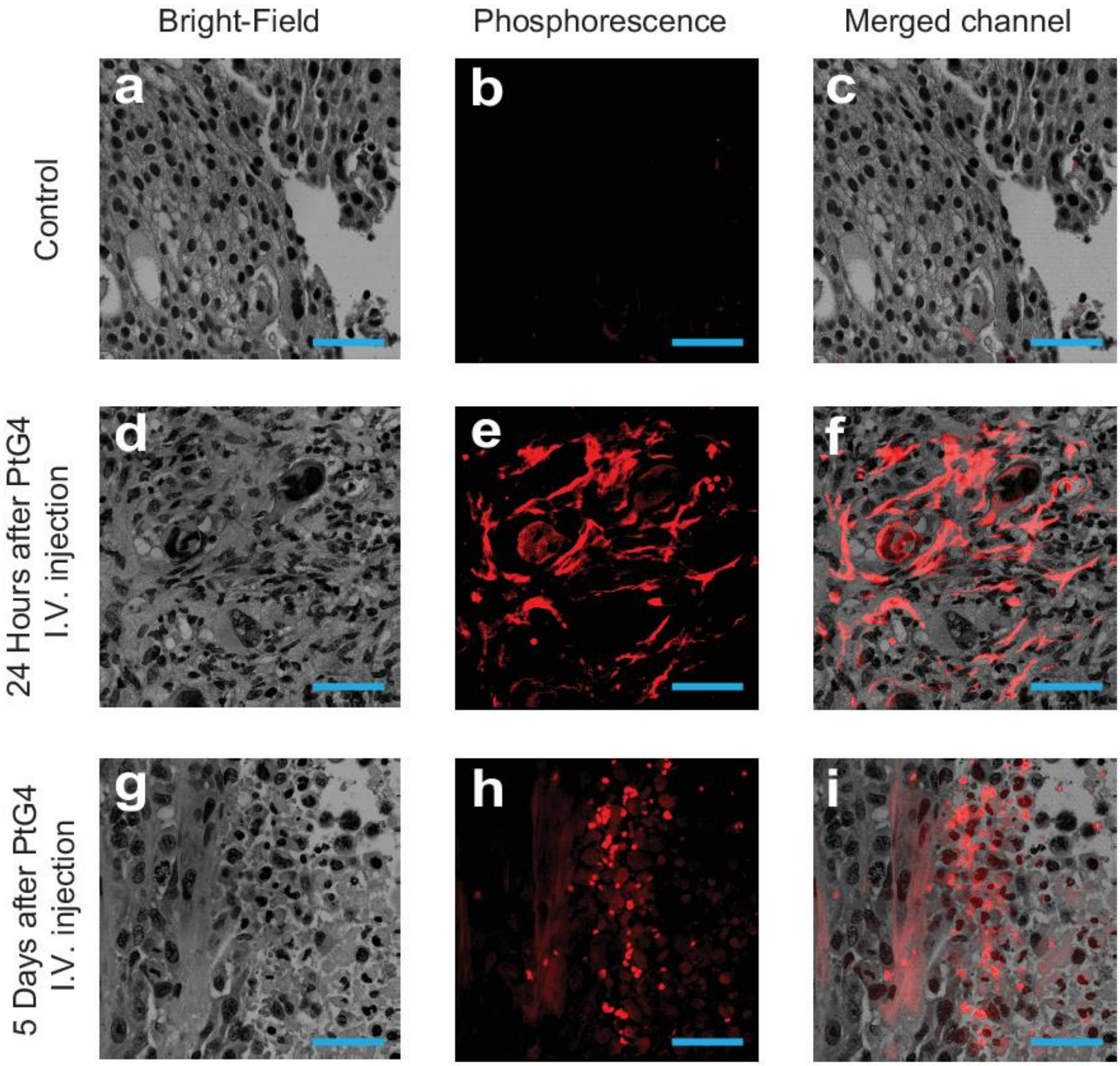

Supplementary Figure 7 Fluorescence microscopy studies of Oxyphor PtG4 localization in tissue. Hematoxylin and eosin (H&E) stained slice of tumor without injection Oxyphor PtG4 (The first row), 24 hours after I.V. injection of Oxyphor PtG4 (The middle row), and 5 days after I.V. injection of Oxyphor PtG4(The third row). (scale bar: 50 μm; Control: the mouse without injection of Oxyphor PtG4).

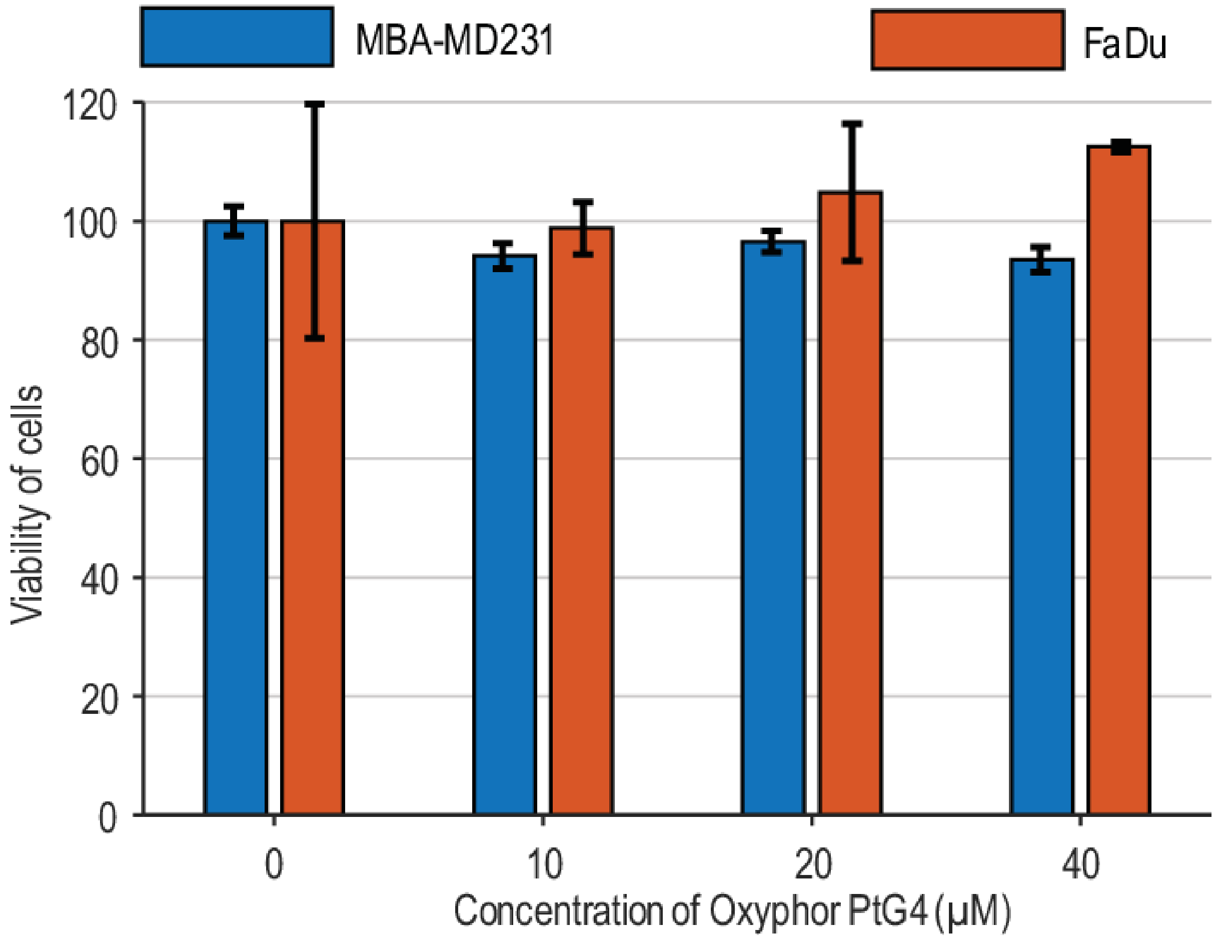

Supplementary Figure 8 Toxicity evaluation using MTT assays of Oxyphor PtG4.

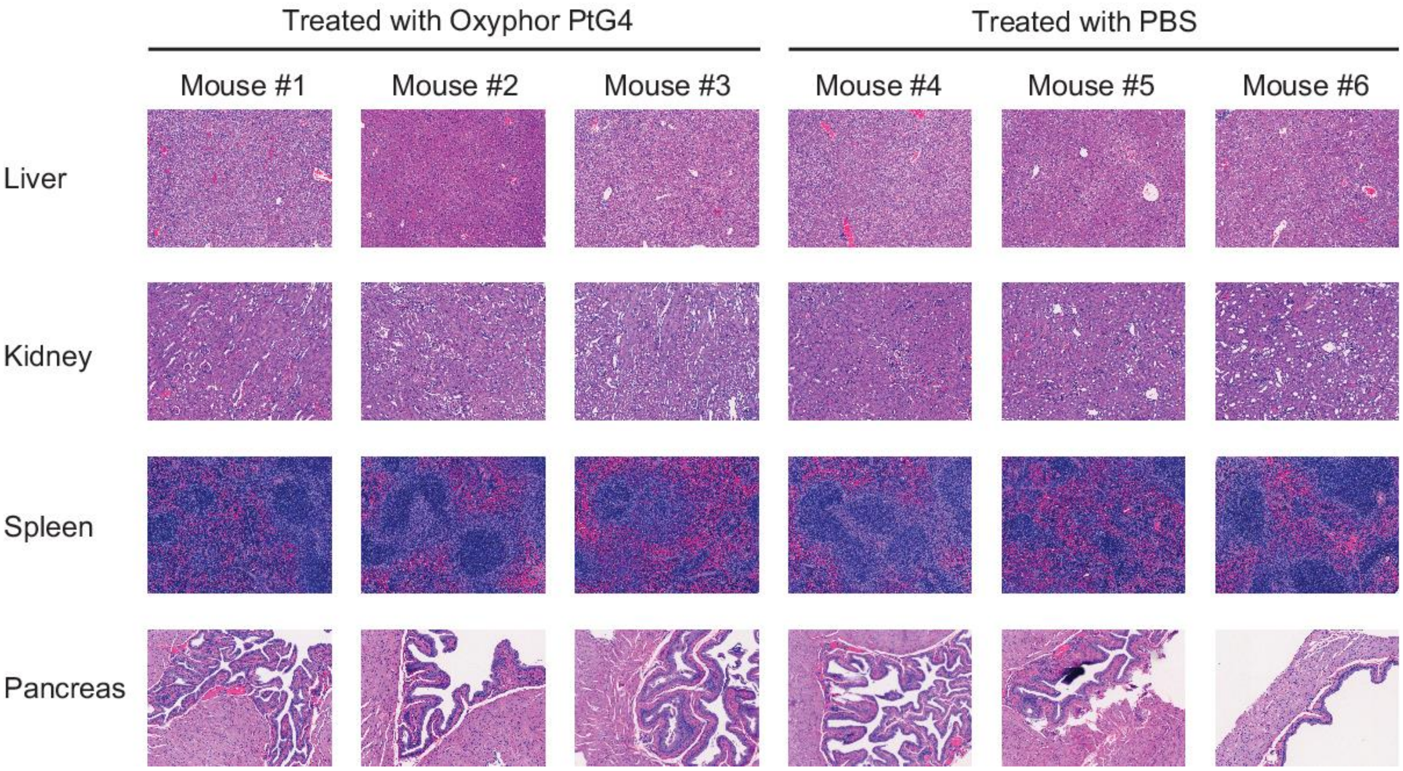

Supplementary Figure 9 Toxicity evaluation using histological H&E staining. Compared to the control mice, the liver, kidney, spleen and pancreas of the Oxyphor PtG4-treated group did not show obvious structural changes.

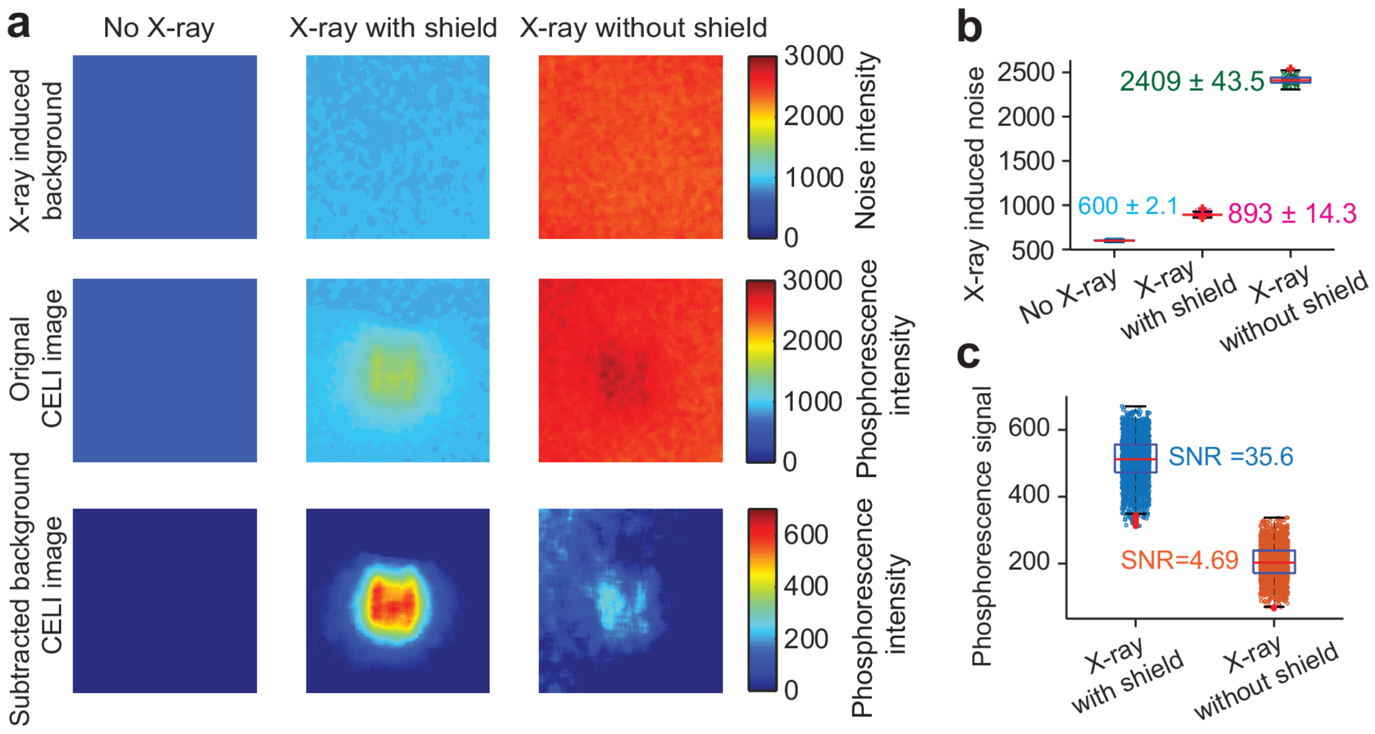

Supplementary Figure 10 Noise suppression for CELI by X-ray block. (a) CELI images obtained under different conditions. The background of the liquid medium (Phosphate buffer saline with 1% intralipid and 1% porcine blood). reached a high level when the X-ray was turned on, while background in the case with X-ray blocking was much lower than that without blocking (upper panel). A tube with solution of Oxyphor PtG4 was placed into the liquid medium at the depth of 5mm and the CELI phosphorescence intensity images were acquired (middle panel). After subtracting the background, the CELI image in the case with X-ray blocking showed phosphorescence of Oxyphor PtG4 (lower panel). (b) Comparison of X-ray induced backgrounds. The data is shown with mean $\pm$SD. (c) Comparison of SNRs, showing that blocking the X-rays afforded a significant improvement in the SNR.

Supplementary Table S1 Comprehensive blood chemistry measurements.

Test	Unit	Oxyphor PtG4			PBS			Range
		#1	#2	#3	#4	#5	#6	
Total Proteins	g/dL	4.9	4.9	4.9	5.1	3.9	4.8	4.5-6.5
Albumin	g/dL	2.9	2.9	2.8	2.9	1.8	2.7	2-3
Globulin	g/dL	2	2	2.1	2.2	2.1	2.1	1.3-3.0
AST (SGOT)	IU/L	73	73	105	127	2397	190	63-154
ALT (SGPT)	IU/L	17	17	24	33	1401	48	32-98
ALP	IU/L	78	78	47	105	90	159	39-299
Total Bilirubin	mg/dL	0.1	0.1	0.1	0.1	0.3	0.2	0.1-0.3
Urea Nitrogen	mg/dL	21	21	26	26	15	21	11-30
Creatine	mg/dL	0.3	0.3	0.3	0.2	0	0.2	0.2-0.8
Phosphorus	mg/dL	6.7	6.7	7.6	7.5	9.6	9.1	6.5-15

Note: AST – aspartate amino transferase, ALT – alanine aminotransferase, ALP – alkaline phosphatase
